# Enhancing the Squareness and Bi-Phase Magnetic Switching of Co_2_FeSi Microwires for Sensing Application

**DOI:** 10.3390/s23115109

**Published:** 2023-05-26

**Authors:** Mohamed Salaheldeen, Asma Wederni, Mihail Ipatov, Valentina Zhukova, Ricardo Lopez Anton, Arcady Zhukov

**Affiliations:** 1 Department of Polymers and Advanced Materials, Faculty of Chemistry, University of the Basque Country, UPV/EHU, 20018 San Sebastián, Spain; 2 Department of Applied Physics I, EIG, University of the Basque Country, UPV/EHU, 20018 San Sebastián, Spain; 3 Physics Department, Faculty of Science, Sohag University, Sohag 82524, Egypt; 4 EHU Quantum Center, University of the Basque Country, UPV/EHU, 20018 San Sebastián, Spain; 5 Department of Applied Physics, Regional Institute for Applied Scientific Research (IRICA), University of Castilla-La Mancha, 13071 Ciudad Real, Spain; 6 IKERBASQUE, Basque Foundation for Science, 48011 Bilbao, Spain

**Keywords:** Heusler alloys, glass-coated microwires, multi-step magnetic behavior, sensing applications

## Abstract

In the current study we have obtained Co_2_FeSi glass-coated microwires with different geometrical aspect ratios, *ρ* = d/D_tot_ (diameter of metallic nucleus, d and total diameter, D_tot_). The structure and magnetic properties are investigated at a wide range of temperatures. XRD analysis illustrates a notable change in the microstructure by increasing the aspect ratio of Co_2_FeSi-glass-coated microwires. The amorphous structure is detected for the sample with the lowest aspect ratio (*ρ* = 0.23), whereas a growth of crystalline structure is observed in the other samples (aspect ratio *ρ* = 0.30 and 0.43). This change in the microstructure properties correlates with dramatic changing in magnetic properties. For the sample with the lowest *ρ*-ratio, non-perfect square loops are obtained with low normalized remanent magnetization. A notable enhancement in the squareness and coercivity are obtained by increasing *ρ*-ratio. Changing the internal stresses strongly affects the microstructure, resulting in a complex magnetic reversal process. The thermomagnetic curves show large irreversibility for the Co_2_FeSi with low *ρ*-ratio. Meanwhile, if we increase the *ρ*-ratio, the sample shows perfect ferromagnetic behavior without irreversibility. The current result illustrates the ability to control the microstructure and magnetic properties of Co_2_FeSi glass-coated microwires by changing only their geometric properties without performing any additional heat treatment. The modification of geometric parameters of Co_2_FeSi glass-coated microwires allows to obtain microwires that exhibit an unusual magnetization behavior that offers opportunities to understand the phenomena of various types of magnetic domain structures, which is essentially helpful for designing sensing devices based on thermal magnetization switching.

## 1. Introduction

The use of ferromagnetic materials in spintronic applications has garnered increasing attention in recent years due to their unique magnetic properties that enable the control and manipulation of spin currents. Among the different types of ferromagnetic materials, micro/nano-structured materials have emerged as promising candidates for enhancing spintronic devices’ performance [1,2,3,4,5,6,7,8,9]. One of the most promising multidisciplinary research fields is spintronics, which enables the creation of the next generation of nano & microdevices with improved processing and memory capability while consuming less power [10]. To address the various required criteria, such as high spin polarization or high Curie temperature, *Tc*, a new generation of materials with multifunction uses has to be created [11]. These Heusler compounds are well suited for spintronic and magneto-electronic applications [12]. Between the advantages of these compounds, we can highlight several ones: good lattice matching with the most typical substrates, *Tc* above room temperature, and the possibility of obtaining close to 100% of spin polarized near the Fermi level [11,12,13,14,15].

In particular, Co_2_-based full-Heusler compounds are among the most promising half-metallic alloys due to their high thermal stability, high Curie temperatures (*Tc* ≈ 1100 K) in bulk form, high magnetic moment (~6 µ_B_/f.u.), and low Gilbert damping constant (*α* = 0.004) [14,16,17]. Additionally, they exhibit interesting transport properties and high magnetic moments. It is noteworthy that these Co_2_-based Heusler alloys present a significant anomalous Hall linked to the enormous Berry curvature associated with their band structure [16,18]. All the precedent evidence why the scientific community is so interested nowadays in Co-based full-Heusler alloys. Hence, these alloys are extensively researched in several configurations: nanoparticles [19], thin films [15,17,20], and nano/microwires [21,22,23,24]. It is relevant to note that the fabrication of Heusler alloys nanoparticles and thin films faces several difficulties for application purposes, including the high cost of preparation methods, chemical composition inhomogeneity, and ease of oxidation [20]. The diffusion of substrate atoms into the film results in the existence of the atomic disorder and phase separations, which are commonly observed [25], in addition to the lattice mismatch between the alloy and the substrate. Furthermore, in order to start the requisite structural ordering, the arc-melted or thin-film-formed Heusler alloys need lengthy, high-temperature annealing procedures [26].

Magnetic wire research has received a lot of interest during the last several decades [27]. The focus is on amorphous magnetic wires, which can exhibit unusual magnetic features such as spontaneous magnetic bistability or the Giant magnetoimpedance phenomenon [27,28]. Several manufacturing processes involving fast solidification can be used to create magnetic wires containing amorphous and/or nanocrystalline phases [27,28]. Nevertheless, only the Taylor-Ulitovsky manufacturing approach allows the preparation of magnetic microwires with the widest diameter range (from 0.2 to 100 µm) [27,28]. Such microwires are composites consisting of metallic nuclei (with 0.2 ≤ d ≤ 100 µm) usually comprised of iron, cobalt, nickel, or their alloys, covered by thin, flexible, and insulating glass (typically Pyrex or Duran) coating (typically with thickness from 0.5 to 10 µm) [27,28]. As a result, the prospective applications of glass-coated microwires in sensing, actuation, and biomedical engineering have been expanded. The insulating and flexible glass coating protects the microwires from oxidation, corrosion, and other environmental factors while simultaneously giving them outstanding mechanical stability. Moreover, the glass layer and the magnetically flexible amorphous metallic nucleus provide high sensitivity to external stimuli including magnetic fields, temperature fluctuations, and mechanical stress [27,28,29,30,31,32,33,34,35]. Such sensitivity is connected to the ferromagnetic origin of the metallic nucleus, which responds to the applied stimulus. Innovative sensors that monitor magnetic fields, temperature, and stress have been developed using glass-coated microwires for a range of applications [27,28,29]. Additionally, they have shown potential characteristics for actuators and in medical applications, including cancer treatment (through magnetic hyperthermia) [29] and medicine administration. Future technological advancements can use glass-coated microwires because of their distinctive combination of properties [27,29].

In this article, we report an attempt to prepare Co_2_FeSi glass-coated microwires with variable geometrical aspect ratios *ρ* = d/D_tot_ (being *d*-diameter of the metallic nucleus and D_tot_—total diameter). The fabrication method was chosen because of the interesting relationship between the magnetic and structural properties in the case of Heusler alloys in the form of glass-coated microwires, coupled with the properties provided by this fabrication method: excellent mechanical properties, insulating behavior, thin and flexible glass-coating, and small dimensionality [27,29,30,31,32,33,34,35]. As a result, we have prepared Co_2_FeSi glass-coated microwires using the Taylor-Ulitovsky procedure, which is detailed previously [27,36,37]. The Taylor-Ulitovsky method, which has been fairly utilized since the 1960s [37], is probably the most used fabrication method to make Heusler alloys glass-coated microwires with a wide variety of geometric characteristics [21,22,24,27,28,29,30,31,32,33,34,35]. The primary benefit of this non-expensive technique is that it allows the production of thin and long (up to several kilometers long) microwires with a wide diameter range (from 0.2 µm up to 100 µm) at a high rate (up to some hundred meters per minute) [36,37,38]. This process is also used to prepare glass-coated microwires with excellent mechanical properties [21,39,40,41]. Additional benefits of glass coating on microwires are better isolation and protection from the surroundings. Moreover, the fact that the glass coating is biocompatible, coupled with the commented properties, make this approach well-suited for biological applications [29,42,43]. Therefore, Heusler microwires of Co_2_FeSi are an interesting material for a broad range of applications and devices. As far as we know, there is no report up to date on the production and structural, mechanical, or magnetic properties of Co_2_FeSi-based glass-covered Heusler microwires with varied *ρ*-ratios, as well as the investigation of its influence on magneto-structure behavior.

## 2. Materials and Methods

Arc melting is a method of manufacturing Co_2_FeSi alloys that involves melting the precursor components together in an electric arc furnace. Typically, the following procedures are used to create Co_2_FeSi alloys by arc melting: (i) preparing the precursor ingredients. The precursor elements for the Co_2_FeSi alloy are weighed and deposited in a graphite crucible, containing cobalt (powder) (99.99%), iron (powder) (99.9%), and silicon (powder) (99.99%) supplied by Technoamorf S.R.L. Co. (Cisineu, Moldavia). (ii) The materials melting. The crucible containing the precursor materials is put in an electric arc furnace, and an electrical current is fed through the materials to start the melting process in a vacuum and argon atmosphere. The furnace temperature is precisely regulated to ensure that the ingredients melt and mix equally. (iii) The cooling and solidification processes. The crucible is withdrawn from the furnace and allowed to cool once the components have melted and combined. The Co_2_FeSi alloy (ingot) is created as the ingredients consolidate. This process was then repeated five times to achieve perfect homogeneity and a homogeneous microstructure. Once the Co_2_FeSi alloy has solidified and formed an ingot, the ingot is used to prepare Co_2_FeSi glass-coated microwires using the Taylor-Ulitovsky process. As described in the introduction, the Taylor-Ulitovsky preparation technique offers significant benefits over alternative procedures for manufacturing glass-coated microwires. One advantage is that it enables the fabrication of microwires with rather thin glass coatings, generally up to a few micrometers thick. This thin insulating coating permits the electrical and magnetic characteristics of the microwire metallic nucleus to be preserved, making the resultant microwires valuable for a wide range of applications. Many prior publications [21,22,24,27,28,29,30,31,32,33,34,35] explain the manufacturing method in detail. To summarize it, a glass capillary was filled with Co_2_FeSi alloy, molten using a high frequency inductor for heating an ingot over its melting temperature. The variation of the speed of wire drawing, alloy temperature, glass tube feed rate and of the rotation of the pick-up bobbin were the parameters used to control the diameter of the metallic nuclei, d_metal_ (µm), and total diameter D_total_ (µm) as explained in detail elsewhere [38]. Finally, the microwire is cooled with a coolant stream to complete the rapid melt quenching process. All geometric parameters of samples investigated in current study are listed in Table 1, also including a similar sample previously studied (in ref. [24]), whose results will be also discussed in the following section.

We used Scanning Electron Microscopy (SEM) and Energy Dispersive X-ray (EDX) (JEOL-6610LV, JEOL Ltd., Tokyo, Japan) to determine the aspect *ρ*-ratio of Co_2_FeSi glass-coated microwires samples and its related nominal chemical composition.

The XRD structure analysis was carried on by using X-ray diffraction (XRD) BRUKER (D8 Advance, Bruker AXS GmbH, Karlsruhe, Germany).

The magnetic behavior was studied in two different ways: hysteresis loops at temperatures between 5 and 350 K, and thermomagnetic curves following three different protocols, zero field cooling (ZFC), field cooling (FC), and field heating (FH) at the low magnetic field (H = 200 Oe). All magnetization curves were measured using a PPMS (Physical Property Magnetic System, Quantum Design Inc., San Diego, CA, USA) vibrating-sample magnetometer at temperatures, T, between 5 and 400 K for ZFC, FC, and FH magnetic curves. For the hysteresis loops, we only focus on the in-plane configuration where the applied magnetic field is parallel to the wire axis. The results are provided in terms of the normalized magnetization, M/M_5K_, where M_5K_ is the magnetic moment obtained at 5 K to avoid misleading of the estimation of the errors in the estimation of the magnetization saturation values.

## 3. Results

### 3.1. Analysis of Chemical and Structural Data

**Figure 1:**
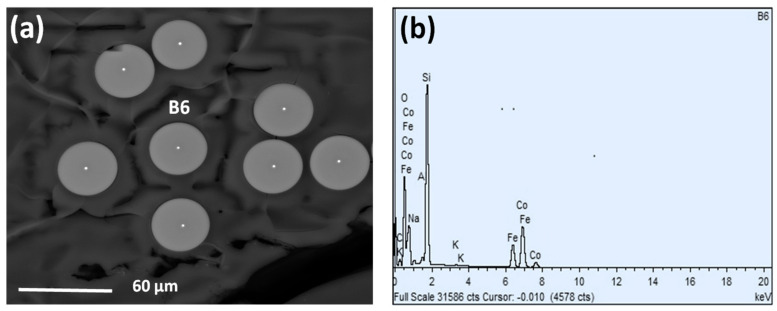
The cross section of selected Co_2_FeSi glass-coated microwires with aspect ratio 0.30 images (**a**) and the chemical composition spectra of EDX of one of the points (**b**).

The geometries (*ρ*-ratios) and chemical compositions of prepared samples are shown in Table 1. The variation of the microwires diameters (d_metal_ and D_total_) is achieved by controlling the drawing rate, alloy temperature, glass tube feed rate, and the receiving bobbin rotation speed [38]. Using the EDX data from Table 1, it was revealed that the metallic nucleus composition differed considerably from the stoichiometric one (Co_2_FeSi). The features of the preparation process, which involved alloy melting and drawing, were the cause of this slight variation. To quantify the difference, we checked the nominal composition for eight sites as illustrated in Figure 1a. An atomic average of Co_44_Fe_23_Si_33_ was used to confirm that the true 2:1 ratio of Co and Fe was applied in all sites. A high Si ratio was found because of the interfacial layer that exists between the metallic nucleus and the glass covering.

In order to study the order state of our produced Co_2_FeSi glass-coated microwires, and to elucidate the effect of the aspect ratio modification on the crystalline structure, XRD structure analysis was carried on by using X-ray diffraction (XRD).

**Figure 2:**
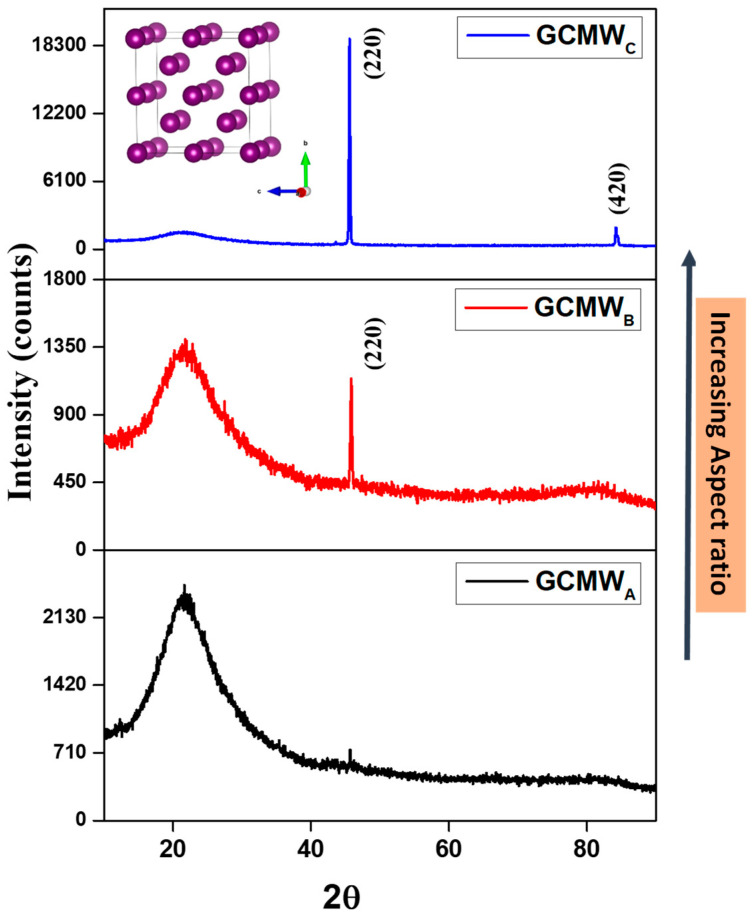
XRD analysis of Co_2_FeSi glass-coated microwires with different aspect ratio measured at room temperature. The inset of Figure 2 indicates the A2-type cubic structure.

As illustrated in Figure 2, the change in the geometric *ρ*-ratio has a strong influence on the structure of Co_2_FeSi glass-coated microwires. For the sample with the lowest *ρ*-ratio, i.e., *ρ* = 0.25, the sample shows an amorphous structure where no crystalline peaks are detected. The wide halo at 2θ = 22.3° is related to the glass coating layer, as reported in our previous works [21,22,24,27,28,29,30,31,32,33,34,35]. By increasing the geometric aspect ratio, a crystalline structure of the metallic nucleus becomes evident with a notable peak at 2θ = 46.2°, attributing to the (220) reflection. Further increase of geometric *ρ*-ratio results in the perfect crystalline structure of samples studied, where the crystalline peak intensity increases and an additional peak appears at 2θ = 85.4°, corresponding to the (422) reflection. The analysis of XRD profiles of the two crystalline Co_2_FeSi samples, i.e., GCMW_C_ (*ρ* = 0.30) and GCMW_B_ (*ρ* = 0.43), indicates an A2 single-phase structure with a small tetragonal distortion (traces of tetragonal martensite phase), and a broadened peak around 22° attributed to an amorphous state for GCMW_C_ and mixed L2_1_ or B2 phases with the amorphous state for GCMW_B_ sample [34,35].

The (220) and (422) reflections in GCMW_C_ sample are split due to some tetragonal distortions of the crystal lattice. A similar phenomenon was seen and discussed elsewhere [44]. It is known that a split in the brag diffraction patterns leads to a small distortion of the crystalline structure [45]. The absence of a (400) peak around 85°, which is expected to be present in the A2 structure, increases the possibility that the crystallites are too fine to be detected by X-rays, as reported elsewhere [46]. In addition, the absence of some peaks can be caused by a similar scattering factor of the constituent elements (Co, Fe, and Si) [47]. Otherwise, according to the theoretical outcomes of Zhang et al., the disordered A2 state is more energetically preferable than those of the ordered L2_1_ or B2 phases [48,49]. Nevertheless, the well-defined and sharp diffraction patterns in this sample (GCMW_C_ sample) indicate a high crystallinity, as compared with the other two XRD spectra. As the development of traces of the secondary phase (tetragonal martensite) can affect magnetic behavior, this will be explored in more detail in the following sections.

We estimated the lattice parameters of the two crystalline Co_2_FeSi glass-coated microwires, and then we employed the Debye-Scherrer’s equation, as presented in our previous work [23], to investigate the microstructure of Co_2_FeSi in greater depth. Using this methodology, we can estimate the average grain size, D_g_, associated with the principal peaks, which is approximately 17.8 m, 37.6 nm, and 45.8 nm for GCMW**, GCMW_B_, and GCMW_C_ of Co_2_FeSi microwires, respectively, as illustrated in Table 2. Thus, D_g_ has a monotonic increase with increasing the aspect ratio.

### 3.2. Magnetic Characterization

#### 3.2.1. Room Temperature Magnetic Properties

**Figure 3:**
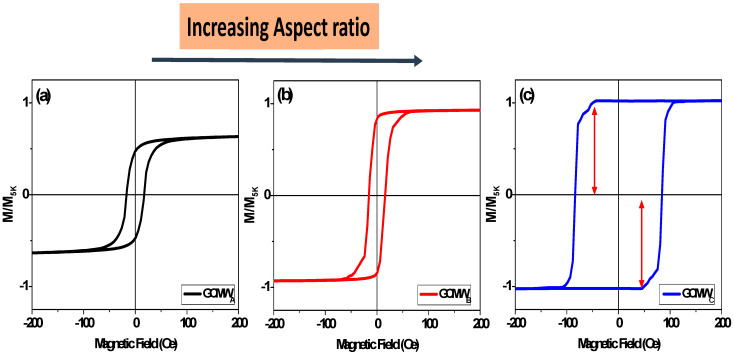
Room temperature hysteresis loops for Co_2_FeSi glass-coated microwires (**a**) GCMW_A_, (**b**) GCMW_B_, and (**c**) GCMW_C_. The arrows in (**c**) pinpoints the multistep magnetic behavior.

**Figure 4:**
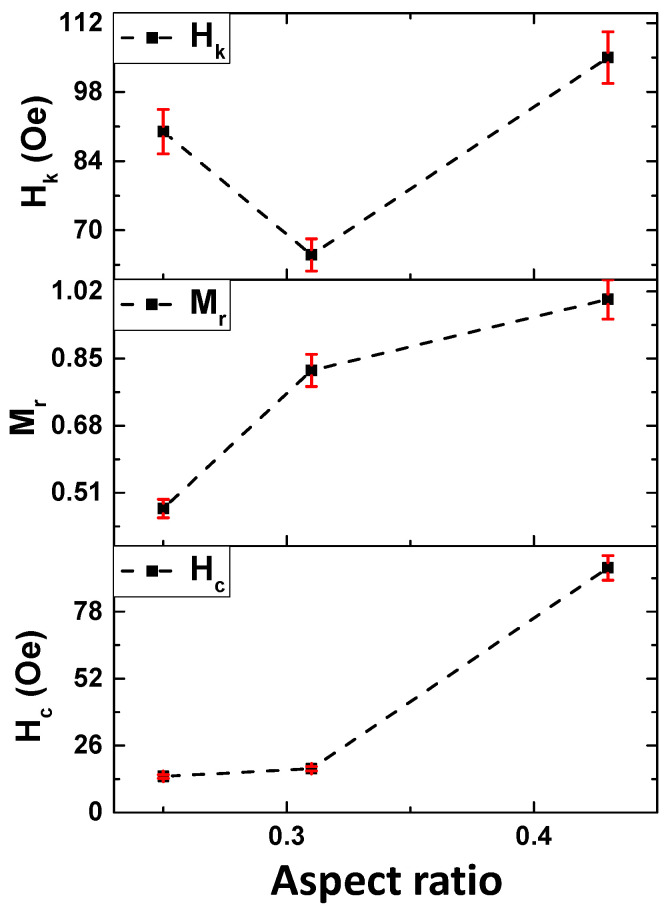
Aspect ratio dependence on coercivity (H_c_), normalized remanence (M_r_), and in –plane anisotropy field (H_k_) of Co_2_FeSi glass-coated microwires (lines for eye guide).

Figure 3 shows the magnetic hysteresis loops of Co_2_FeSi-glass-coated microwires with different *ρ*-ratios, obtained at room temperature with an applied magnetic field parallel to the microwire axis. All samples exhibit typical ferromagnetic behaviour, due to the high Curie point of Co_2_FeSi alloy greater than 1100 K [46]. The sample with a low *ρ*-ratio exhibits soft magnetic properties with coercivity, H_c_, around 14 Oe, and a non-square hysteresis loop shape (Figure 3a). However, the sample with the largest *ρ*-ratio shows almost perfectly square hysteresis loops with higher H_c_ (about 87 Oe), than Co_2_FeSi with a low *ρ*-ratio (see Figure 3b,c). In addition, the hysteresis loop shows multistep magnetic behavior (indicated with arrows in Figure 3c). The almost square hysteresis loops for the GCMW_C_ microwire with normalized remanent, M_r_, near 0.96 indicates the axial character of magnetic anisotropy with the easy axis of magnetization along the direction of the applied magnetic field. Thus, the increase in *ρ*-ratio affects the magnetocrystalline anisotropy, and its direction has the same direction of (220) and (420), as illustrated in the structural section. However, in the sample GCMW_B_ with a crystalline structure, non-perfectly square loops are observed. Such change in the hysteresis loop shape must be related to the presence of a considerable amount of amorphous phase beside the disordered B2 or little-ordered L2_1_ structures. In our previous work at the same alloys, but with a low *ρ*-ratio (*ρ* = 0.26), the enhancement of the magnetocrystalline anisotropy, the squareness, and coercivity of Co_2_FeSi glass-coated microwires after annealing was observed [21,22,24]. As we illustrated in our previous work, the two main factors affecting the magnetic anisotropy behavior in Heusler-based glass-coated microwires are uniaxial magnetic anisotropy and cubic magnetocrystalline anisotropy [21,22,24]. By increasing the *ρ*-ratio, an enhancement in the crystalline phase content correlates with the magnetic property modification, i.e., the main factor controlling the magnetic anisotropy is the cubic magnetocrystalline anisotropy. Unfortunately, currently, we are not able to measure this type of anisotropy experimentally, but the perfectly square loop indicates its strong effect on the GCMW_B_ and GCMW_C_ samples. As seen in Figure 4, the GCMWc sample shows the highest anisotropy field H_k_, coercivity H_c_, and normalized remnant M_r_. The odd behavior of H_k_ is likely related to the big amorphous phase present in GCMW_A_ and GCMW_B_ samples, as the behavior of Hc for these two samples is also quite similar (there is not a decrease, as in the case of the H_k_, but the values are almost the same) and maybe the growth of the crystalline structure eases initially the reduction of the anisotropy field. In addition, the different types of microstructures (L21, B2, and A2) can also strongly affect the H_k_ behavior.

#### 3.2.2. Thermomagnetic Properties

**Figure 5:**
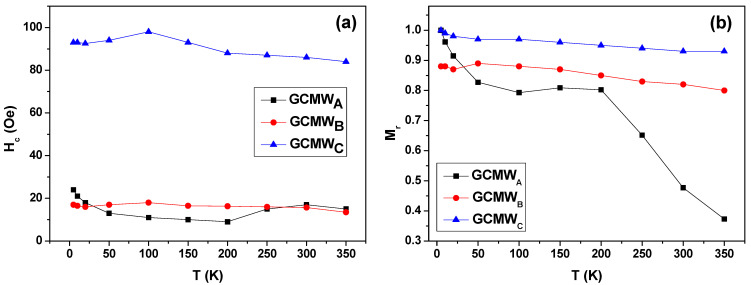
Temperature dependence of the coercivity (**a**) and normalized remanence (**b**) of Co_2_FeSi glass-coated microwires with different aspect ratio (lines for eye guide). The error bar is as big or smaller than the size of the symbols.

It is worth noting that the ferromagnetic materials temperature stability is a crucial characteristic for their possible applications in spintronic and sensing devices. Hence, for a wide range of measurement temperatures, 5–350 K, we investigated the magnetic behavior of Co_2_FeSi glass-coated microwires with different *ρ*-ratios. The shape of the loops follows the same trend observed at room temperature: non-square for the GCMW_A_ sample, quite square for the GCMW_B_ one, and almost square for the GCMW_C_ one (loops not shown). In Figure 5, the evolution of H_c_ and M_r_ with the temperature is shown. This behavior demonstrates that for the GCMW_C_ sample, cubic magnetocrystalline anisotropy prevails up to 350 K.

By analyzing the hystersis loops measured at temperature range, 5–350 K of Co_2_FeSi glass-coated microwires with different *ρ*-ratio, an interesting magnetic behavior is found for both the temperature dependence of H_c_ and of the normalized remanence, M_r_. GCMW_C_ sample shows the highest value of the coercivity at the all measuring range of temperature range, with an average value of H_c_ six times higher than those of the GCMW**, GCMW_A_ and GCMW_B_ samples. GCMW**, GCMW_A_, and GCMW_B_ samples show quite similar values of the H_c_ where the difference between the average value of coercivity is about 2 Oe. By estimating the differences in the coercivity (ΔHc) between the maximum value of coercivity (H_c (max)_) and the lowest value of the coercivity (H_c (min)_) for all samples, we pretend to show its stability with temperature. The samples with a clear crystalline phase, GCMW**, GCMW_B,_ and GCMW_C_ samples, show higher temperature stability than the amorphous GCMW_A_ sample. Hence, the ΔHc is 11 Oe, 3.5, and 9 Oe for GCMW**, GCMW_B,_ and GCMW_C_ samples, respectively, whereas ΔHc is 15 Oe for the GCMW_A_ one. The magnetic stability is clearer in the case of Mr tendency with the temperature of Co_2_FeSi glass-coated microwires with different *ρ*-ratios. As shown in Figure 5b, both GCMW_B_ and GCMW_C_ samples show high stability with temperature, with ΔM_r_ 0.05 and 0.06, respectively (see Table 3). Meanwhile, the behavior of Mr of GCMW_A_ is rather different, compared to the other samples with higher *ρ*-ratios, where a monotonic increase with decreasing the temperature has been observed.

**Figure 6:**
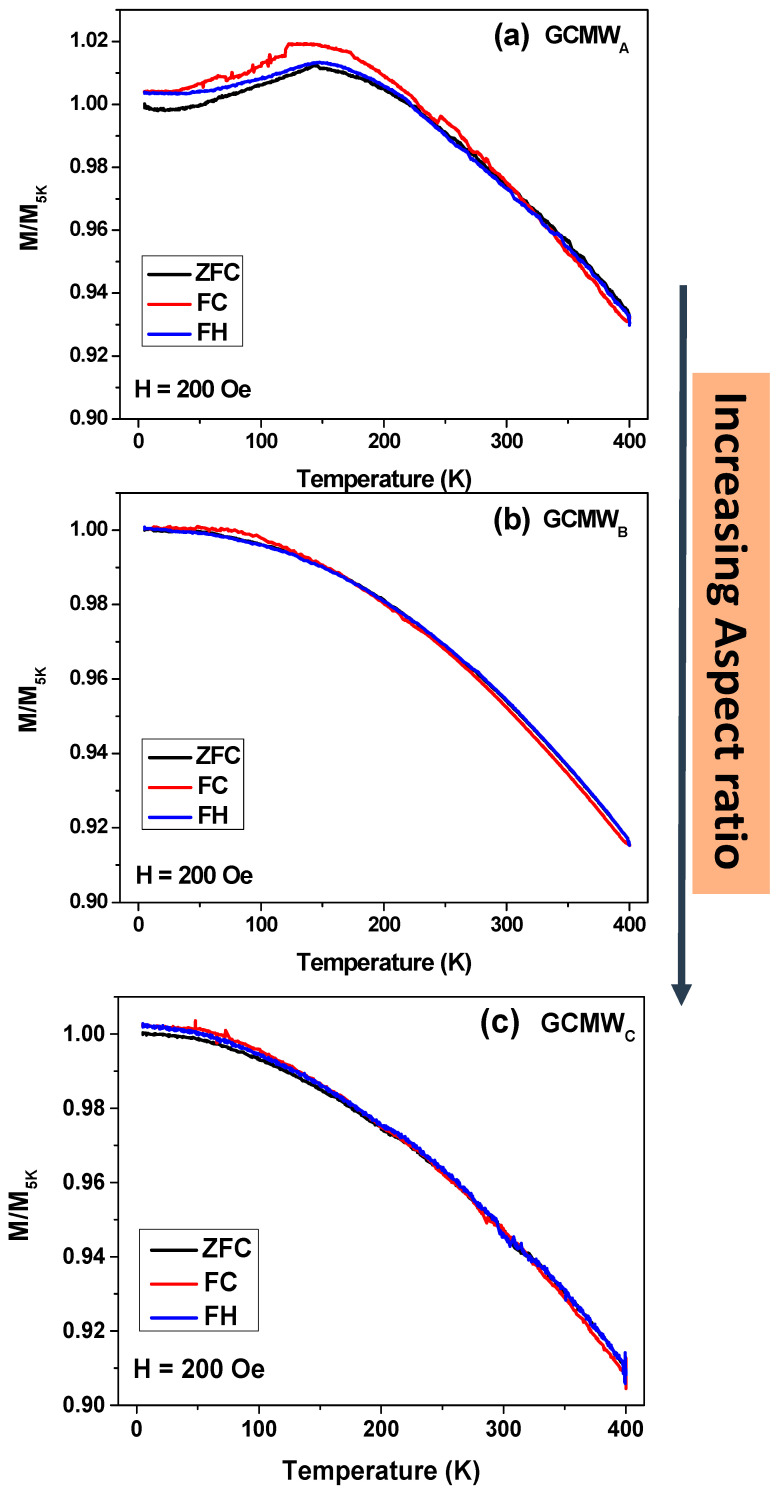
Temperature dependence of magnetization measured for Co_2_FeSi glass-coated microwires (**a**) GCMW_A_, (**b**) GCMW_B_, and (**c**) GCMW_C_ with applied external magnetic field 200 Oe.

Figure 6 shows the complete thermomagnetic behavior of Co_2_FeSi glass-coated microwires with different *ρ*-ratios. We performed the ZFC, FC, and FH magnetic temperature dependence to check any possible phase transition. Thus, the measurements were performed at a low magnetic field of 200 Oe. For the GCMW_A_ sample, the ZFC, FC, and FH magnetizations curves show non-homogonous behavior, besides an irreversible magnetic behavior at T = 150 K. Such irreversibility has been observed in our previous work dealing with Co_2_FeSi-based glass-coated microwires with aspect ratio *ρ* = 0.25, i.e., GCMW**, (see [21,22,24]). In this work, we have illustrated that the irreversibility is enhanced by performing annealing at 873 K and 973 K for 1 h. The induced martensitic transition and the change in the internal stresses associated with the glass-covering layer with temperature allow to control the irreversibility behavior. The interesting point for Co2FeSi glass-coated microwires with the lowest *ρ*-ratio, i.e., *ρ* = 0.23, is that its blocking temperature is observed at T = 150 K, such as the Co2FeSi glass-coated microwires with *ρ* = 0.25, i.e., GCMW** [21,22,24]. For the GCMW_A_ sample, which is totally in an amorphous state (see Figure 2), the main reason for the irreversibility behavior is the strong internal stress induced by the glass covering layer. For GCMW_B_ and GCMW_C_ samples, with increasing *ρ*-ratio, the irreversibility behavior disappeared, and the usual ferromagnetic behavior is observed with homogenous ZFC, FC, and FH magnetic curves. The homogenous magnetization curves are due to the induced crystal structure with A2-type and B2 or L2_1_ cubic structure for GCMW_C_ and GCMW_B_, respectively.

We believe that increasing the *ρ*-ratio of Co_2_FeSi glass-coating microwires affects recrystallization, atomic ordering, and stress reduction. Furthermore, for samples with a high *ρ*-ratio, the induced L2_1_/B2 and A2 cubic structure types generate a strong magneto crystalline anisotropy, explaining the behavior of magnetic properties such as H_c_, M_r_, H_k_, and thermomagnetic curves with temperature. In fact, as shown in several previous publication, the internal stress values are affected by the *ρ*-ratio: the lower the *ρ*-ratio, the higher the internal stresses related to the presence of the glass-coating [50,51,52]. On the other hand, the glass-coating thermal conductivity can affect the quenching rate of the metallic nucleus: a lower quenching rate must be at the origin of higher crystallinity of the microwires with relatively thick glass-coating [27]. The aforementioned internal stresses together with the samples’ microstructure (average grain size) can affect the hysteresis loops. Additionally, the presence of the interfacial layer between the metallic nucleus and glass-coating, reported by us earlier can affect the saturation magnetization [53]. Such influence of the interfacial layer must be more appreciable for the thinner d_metal_-values.

In summary, Co_2_FeSi glass-coated microwires are an excellent candidate for a wide range of industrial applications, especially for sensors, due to their perfect squared loops at a wide temperature range and homogenous thermomagnetic behavior with temperature.

## 4. Conclusions

In summary, we have fabricated Co_2_FeSi-glass-coated microwires with different geometrical aspect ratios. A strong influence of the geometric aspect ratio on the magnetic and structural properties is observed and discussed. The increase in the aspect ratio correlates with the increasing degree of crystallinity. Promising coercivity and normalized remnant stability with temperature are found for Co_2_FeSi glass-coated microwires with a high aspect ratio. For the sample with the lowest aspect ratio, the thermomagnetic curves show large irreversibility with a blocking temperature of T = 150 K. The induced crystal structure of the A2-type gives rise to a high cubic magnetocrystalline anisotropy that controls the magnetic behavior of Co_2_FeSi glass-coated microwires and makes it a suitable candidate for magnetic sensing.

## Figures and Tables

**Table 1:** The geometrical parameters d_metal_ (µm), D_total_ (µm), aspect ratio, and average (Av.) of atomic percentage of Co, Fe, and Si elemental composition in Co_2_FeSi glass-coated microwires.

Sample	d_metal_ (µm)	D_total_ (µm)	Aspect Ratio (*ρ*)	Chemical Composition
GCMW_A_	5.1 ± 0.1	22.2 ± 0.1	0.23 ± 0.01	Co_44_Fe_23_Si_33_
GCMW**	4.4 ± 0.1	17.6 ± 0.1	0.25 ± 0.01	Co_44_Fe_23_Si_33_
GCMW_B_	6.4 ± 0.1	21.3 ± 0.1	0.30 ± 0.01	Co_44_Fe_23_Si_33_
GCMW_C_	7.7 ± 0.1	17.9 ± 0.1	0.43 ± 0.01	Co_44_Fe_23_Si_33_

GCMW**: Co_2_FeSi glass-coated microwires with (*ρ* = 0.25) [24].

**Table 2:** The average grain size and lattice parameters of Co_2_FeSi glass-coated microwires with different aspect ratios.

Sample	Average Grain Size (nm)	Lattice Parameters (Å)
GCMW_A_	-	-
GCMW**	17.8 ± 0.1	5.64 ± 0.01
GCMW_B_	37.6 ± 0.1	5.63 ± 0.01
GCMW_C_	45.8 ± 0.1	2.81 ± 0.01

GCMW**: Co_2_FeSi glass-coated microwires with (*ρ* = 0.25) [24].

**Table 3:** The geometrical parameters and average (Av.) of Co_2_FeSi glass-coated microwires with different aspect ratios.

Sample	ΔH_c_ (H_c (max)_ − H_c (min)_)	ΔM_r_ (M_r (max)_ − M_r (min)_)
GCMW_A_	15 ± 2 Oe	0.7 ± 0.1
GCMW**	11 ± 1 Oe	0.6 ± 0.1
GCMW_B_	3.5 ± 0.5 Oe	0.06 ± 0.01
GCMW_C_	9 ± 2 Oe	0.05 ± 0.01

GCMW**: Co_2_FeSi glass-coated microwires with (*ρ* = 0.25) [24].

## Data Availability

Not applicable.
